# Effect of Sitagliptin and Metformin on Prediabetes Progression to Type 2 Diabetes - A Randomized, Double-Blind, Double-Arm, Multicenter Clinical Trial: Protocol for the Sitagliptin and Metformin in PreDiabetes (SiMePreD) Study

**DOI:** 10.2196/resprot.5073

**Published:** 2016-08-04

**Authors:** Poobalan Naidoo, Jeffrey Wing, Virendra Rambiritch

**Affiliations:** ^1^ University of Witwatersrand, Faculty of Health Sciences, Department of Endocrinology Gauteng South Africa; ^2^ University of KwaZulu Natal, Faculty of Health Sciences Durban South Africa

**Keywords:** primary prevention, type 2 diabetes mellitus, prediabetes, dipeptidyl peptidase-IV

## Abstract

**Background:**

The high prevalence and incidence of type 2 diabetes mellitus (DM), and its associated morbidity and mortality, has prompted growing international interest and effort in the primary prevention of this disease. Primary prevention is possible since type 2 DM is preceded by prediabetes, offering a window opportunity to treat patients, and prevent the emergence of advanced disease. Sitagliptin is an oral dipeptidyl peptidase-IV inhibitor that preserves existing beta cell function and increases beta cell mass. These two effects have been demonstrated both in vitro and in animal studies, and current clinical data show that sitagliptin is safe. Metformin, a biguanide, reduces insulin resistance and inhibits hepatic gluconeogenesis, and has an excellent safety profile. The combination of metformin and sitagliptin, targeting both characteristics of prediabetes (insulin resistance and progressive beta cell degeneration), may potentially slow or halt the progression from prediabetes to type 2 DM. This paper describes the rationale and design of the Sitagliptin and Metformin in PreDiabetes (SiMePreD) study.

**Objective:**

The aim of this study is to determine the effect of sitagliptin and metformin on progression from prediabetes to type 2 DM. The objectives of the study are to determine the effects of metformin and placebo on glycemic endpoints, the effects of sitagliptin and metformin on glycemic endpoints, the effects of metformin and placebo on incidence of cardiovascular disease and death, and the effects of sitagliptin and metformin on incidence of cardiovascular disease and death.

**Methods:**

This is a randomized, double-blind, multicenter clinical study that will determine if the combination of metformin and sitagliptin is effective in preventing the progression from prediabetes to type 2 DM. The study will contain two arms (metformin/sitagliptin and metformin/placebo). Primary endpoints include the number of subjects progressing from prediabetes to type 2 DM, the number of cardiovascular events, and the number of deaths. The planned duration of the study is five years, and 410 subjects will be included in each group. Data analyses will include clinically relevant measures (eg, numbers needed to treat and numbers needed to harm) and will be performed according to the intention-to-treat principle.

**Results:**

This study is currently in the process of acquiring research funding.

**Conclusions:**

The SiMePreD study is the first study to investigate the utility of sitagliptin in combination with metformin for the primary prevention of type 2 DM.

## Introduction

Diabetes mellitus (DM) is a metabolic disorder characterized by chronic hyperglycemia with disturbances of carbohydrate, lipid, and protein metabolism, resulting from defects in insulin secretion, insulin action, or both [1]. The World Health Organization estimates that between 120 and 140 million people suffer from DM worldwide, and that this number could double by the year 2025 [2]. Most of the increase will occur in developing countries and will be due to population aging, diet, obesity, and a sedentary lifestyle [2,3]. DM is associated with a significant decrease in life expectancy and is a risk equivalent to established coronary artery disease [4]. In patients who develop DM, cardiovascular morbidity and mortality are increased by 2-to-6 fold [5,6].

Prediabetes is a metabolic condition characterized by insulin resistance and primary or secondary beta cell dysfunction, which increases the risk of type 2 DM [7]. The American Diabetes Association defines prediabetes as either impaired glucose tolerance (IGT; 2-hour postprandial glucose of 7.8-11.0 mmol/L) or impaired fasting blood glucose (FBG; value of 5.6-6.9 mmoI/L), or both [1]. Risk factors for prediabetes include family history of diabetes, excess body weight (particularly abdominal adiposity), age >45 years, gestational diabetes, high birth weight children, certain ethnic groups, hypertension, and physical inactivity [8]. Glucose levels above the normal, but below the threshold diagnostic for diabetes, are associated with a substantially increased risk of developing cardiovascular disease and death [9,10].

Subjects that eventually develop type 2 DM progress from normal glucose tolerance to IGT, and finally to type 2 DM [11]. Edelstein et al [12] investigated the predictors of progression from IGT to type 2 DM in data from six prospective studies. This study concluded that individuals with IGT have an increased risk of developing type 2 DM.

The progression of the disease is related to deterioration in beta cell function and increased insulin resistance [1]. IGT precedes type 2 DM, providing an attractive target for intervention, and thus entertains the possibility of slowing down or preventing progression to type 2 DM.

The growing prevalence of type 2 DM and its high associated mortality and morbidity make the prevention of this disease an important public health intervention [13]. Patients with type 2 DM and those with prediabetes are at an increased risk for the development of cardiovascular diseases [14]. Halting the progression from IGT to type 2 DM is therefore an important health intervention strategy.

Interventions to delay or even prevent type 2 DM have the potential to improve the health of populations, and reduce health care costs associated with the management and prevention of diabetic complications [15]. Various interventions have been used to prevent or delay the progression from IGT to type 2 DM [16], including pharmacological agents, lifestyle modification (LSM), and herbal remedies. Pharmacological interventions have included oral antidiabetic drugs and antiobesity drugs.

### Nonpharmacological and Pharmacological Interventions for Prediabetes

During the conception of the Sitagliptin and Metformin in PreDiabetes (SiMePreD) study, we discussed various interventions for prediabetes. A summary of selected clinical trials on prediabetes is presented below. The rationale for the choice of drugs for the SiMePreD study will be detailed in the discussion section.

#### Nonpharmacological Interventions

##### Lifestyle Modification and the Prevention of Diabetes Mellitus

Three randomized studies [17-19] have demonstrated a positive effect of LSM on DM prevention. The Da Qing IGT and Diabetes Study [17] screened 110,660 men and women for IGT and DM, of whom 577 had IGT (as per World Health Organization criteria for IGT). Subjects with IGT were randomized either to a control group or to one of three active treatment groups: diet only, exercise only, or diet-plus-exercise. Follow-up evaluation examinations were conducted at 2-year intervals over a 6-year period to identify subjects who developed type 2 DM. The cumulative incidence of diabetes at 6 years was 67.7% (95% CI 59.8-75.2) in the control group compared with 43.8% (95% CI 35.5-52.3) in the diet group, and 41.1% (95% CI 33.4-49.4) in the diet-plus-exercise group (*P*<0.05). The relative decrease in rate of development of diabetes in the active treatment groups was similar when subjects were stratified as lean or overweight. After adjustment for differences in baseline body mass index (BMI) and fasting glucose, the diet, exercise, and diet-plus-exercise interventions were associated with 31% (*P*<0.03), 46% (*P*<0.05), and 42% (*P*<0.05) reductions in risk of developing diabetes, respectively. The study demonstrated that the diet alone, exercise alone, or the combination of the two interventions resulted in the reduced incidence of DM over a 6-year period in subjects with IGT.

The Finish Diabetes Prevention Study [18] randomly assigned 522 middle-aged, overweight subjects (172 men and 350 women; mean age 55 years; mean BMI 31 kg/m^2^) with IGT to either the intervention group (individualized counseling aimed at reducing weight and total intake of saturated fat, and increasing intake of fiber and physical activity) or the control group. An oral glucose-tolerance test was performed annually; the diagnosis of diabetes was confirmed by a second test. The mean duration of the follow-up was 3.2 years. The cumulative incidence of diabetes after four years was 11% (95% CI 6-15%) in the intervention group and 23% (95% CI 17-29%) in the control group. The risk reduction was 58% (*P*<0.001) in the intervention group. The reduction in the incidence of diabetes was directly associated with changes in lifestyle.

The Diabetes Prevention Program Research Group [19] randomly assigned 3234 nondiabetic subjects with elevated fasting and postload plasma glucose concentrations to placebo, metformin treatment, or an LSM program. The average follow-up was 2.8 years. The incidence of diabetes was 11.0, 7.8, and 4.8 cases per 100 person years in the placebo, metformin, and lifestyle groups, respectively. The lifestyle intervention reduced DM incidence by 58 percent (95% CI 48-66%) and metformin reduced incidence by 31 percent (95% CI 17-43%), as compared with placebo. To prevent one case of diabetes during a period of three years, 6.9 persons would have to participate in the lifestyle intervention program, and 13.9 would have to receive metformin. The American Diabetes Association [20] recommends exercise as a component of DM prevention. Diet and exercise interventions improve insulin resistance and decreases the incidence of diabetes and cardiovascular events, although long term weight loss is difficult to maintain [17,18,21,22].

#### Pharmacological Interventions

##### Metformin and the Prevention of Diabetes Mellitus

Metformin has been studied for more than 50 years and has been shown to be safe, even with long term use [23,24]. Observational and randomized studies have shown that metformin is the most effective oral hypoglycemic agent for reducing cardiovascular morbidity and mortality in patients with DM, and is considered first line treatment [24-27]. A meta-analysis of metformin treatment in persons at risk for DM has concluded that metformin treatment results in substantial reductions in the development of type 2 DM (odds ratio 0.6 [0.5-0.8]) [28].

Ramachandran et al [22] investigated the effect of LSM and metformin on the prevention of type 2 DM in Asian Indian subjects with IGT. Study subjects (n=531) with IGT were randomly allocated to four groups: control, advice on LSM, metformin alone, and LSM combined with metformin. The primary outcome measure was type 2 DM. The median follow-up period was 30 months, and the 3-year cumulative incidences of diabetes ranged between 39.5-55.0%.

The relative risk reduction was 28.5% with LSM (95% CI 20.5-37.3, *P*=0.018), 26.4% with metformin (95% CI 19.1-35.1, *P*=0.029) and 28.2% with LSM and metformin (95% CI 20.3-37.0, *P*=0.022), as compared to the control group. To prevent one case of diabetes, 6.9 persons would need to be treated with metformin, and 6.5 persons for LSM combined with metformin. The investigators concluded that both LSM and metformin significantly reduced the incidence of diabetes in Asian Indians with IGT. The lifestyle intervention was more effective than metformin. However, the intense LSM group had to endure a program that is unlikely to be sustained in real world settings.

##### Thiazolidinediones and the Prevention of Diabetes Mellitus

The Diabetes Reduction Assessment with Ramipril and Rosiglitazone Medication trial [29] enrolled 5269 adults with impaired fasting glucose or IGT, or both. These study subjects were followed for a median of 3 years and the primary outcome was a composite of incident diabetes or death. Three hundred and six (11.6%) individuals given rosiglitazone and 686 (26.0%) given placebo developed the composite primary outcome (hazard ratio 0.40, 95% CI 0.35-0.46; *P*<0.001); 1330 (50.5%) individuals in the rosiglitazone group and 798 (30.3%) in the placebo group became normoglycemic (median 1.71, 1.57-1.87; *P*<0.001). Fourteen (0.5%) participants in the rosiglitazone group and two (0.1%) in the placebo group developed heart failure (*P*=0.01). Rosiglitazone was associated with greater incidence of heart failure.

The Troglitazone in Prevention of Diabetes (TRIPOD) study, a randomized double-blind study, investigated the effect of troglitazone (400 mg/day; n=133) versus placebo (n=133) in women with previous gestational diabetes [30]. The average annual diabetes incidence rates in women who returned for follow-up were 12.1% in the placebo group and 5.5% in the troglitazone group. There was a significantly lower cumulative incidence of diabetes in the troglitazone group. The hazard ratio for diabetes was 0.45 (95% CI 0.25-0.83) and was unchanged (hazard ratio=0.44) by adjustment for differences in baseline and on-trial. The hazard ratio for diabetes in the troglitazone group was 0.50 (95% CI 0.28-0.89) and 0.44 with adjustment for differences in baseline and on-trial characteristics. Thus, troglitazone reduced the incidence of diabetes in women who returned for follow-up by at least 50%.

The Pioglitazone in Prevention of Diabetes (PIPOD) study [31] was an open-label observational study to determine the effects of pioglitazone in women with prior gestational diabetes who had completed the TRIPOD study. The PIPOD study consisted of 3 years of drug treatment and 6 months of postdrug washout. The average dropout rate for the study period was 9.6% (n=24). Of the 24 patients that did not complete the study, 19 women moved away from the study area, 10 withdrew consent for personal reasons, and none of these patients had diabetes. Five women failed to come for scheduled appointments either immediately after enrolment (n=3) or after a period of active participation (n=2), and attempts to contact them failed, so their diabetes status at the time of drop out was unknown. Incidence rates of diabetes were calculated from 86 women (42 from the active treatment arm of the TRIPOP study). Eleven participants had diabetes at one or more oral glucose tolerance tests during a median of 35.9 months of pioglitazone treatment. No new cases of diabetes were observed during the post-drug wash-out, which lasted a median of 5.7 months.

Average annual incidence rates of diabetes were 5.2% during pioglitazone treatment and 4.6% during the entire observation period, including the postdrug washout. The final cumulative incidence of diabetes during treatment and postdrug follow-up was 17%. These rates were similar to analogous rates observed during a median of 31 (standard deviation [SD] 8) months of troglitazone treatment and posttrial washout in the TRIPOD study (5.7% and 25% per year, respectively) and lower than rates observed during a median of 28 (SD 8) months of placebo treatment and posttrial washout in the TRIPOD study (13.1% and 52% per year, respectively).

##### Combination of Thiazolidinediones and Biguanides in Diabetes Prevention

Thiazolidinediones and biguanides have different modes of pharmacological action. Metformin inhibits hepatic glucose production, while thiazolidinediones produce a greater effect on peripheral glucose uptake. Basal insulin concentrations are not raised with metformin or thiazolidinediones, thus there is a minimal risk of hypoglycemia, and metformin can reduce the weight gain associated with thiazolidinediones [32]. The combination of biguanides and thiazolidinediones has been used to treat type 2 DM [33,34]. Fonseca et al [33] investigated the effect of metformin and rosiglitazone combination therapy in patients with type 2 DM using a randomized, double-blind, placebo controlled trial. The study concluded that combination treatment with once-daily metformin/rosiglitazone improved glycemic control, insulin sensitivity, and beta cell function more effectively than treatment with metformin alone. Dose-dependent increases in body weight and total and low-density lipoprotein cholesterol levels were observed (*P*<0.001) for both rosiglitazone groups versus placebo. The proportion of patients reporting adverse events was comparable across all groups.

Rosenstock et al [34,35] compared treatment with rosiglitazone/metformin fixed-dose combination therapy with monotherapy of either rosiglitazone or metformin in patients with uncontrolled type 2 DM. This study found that the rosiglitazone/metformin therapy achieved significant reductions in glycosylated hemoglobin (HbA1c) and fasting plasma glucose compared with either drug used as monotherapy.

The Canadian Normoglycemia Outcomes Evaluation (CANOE) study [35], a randomized double-blind controlled trial with a median duration of 3.9 years, investigated whether low dose combination therapy with rosiglitazone and metformin would prevent type 2 DM. One hundred and three subjects with IGT were assigned to rosiglitazone/metformin, and 104 to placebo. The CANOE study demonstrated that low dose therapy with rosiglitazone/metformin effectively prevented the onset of DM, and 4.0 persons would need to be treated with this combination to prevent one case of DM. A significant increase in diarrhea was observed in the active arm compared to placebo (16% vs 6%, *P*=0.0253).

##### Sitagliptin and the Potential in Diabetes Prevention

Dipeptidyl peptidase-IV (DPP-IV) inhibitors are a new class of antidiabetic drugs. These drugs enhance the body’s ability to regulate blood glucose by increasing the active levels of incretins, glucagon-like peptide 1 (GLP-1) and glucose dependent insulinotropic peptide (GIP) [36]. Sitagliptin is a DPP-IV inhibitor that increases insulin release and decreases glucagon levels by preventing the deactivation of GLP-1 and GIP [37]. Sustained receptor activation is associated with insulin biosynthesis and stimulation of beta cell proliferation [37].

Cumulative clinical trials with sitagliptin have enrolled >2600 patients with type 2 DM [36]. In these trials, study subjects received sitagliptin in doses of 100 mg/day for at least 12 weeks; >1000 patients received sitagliptin in doses of 100 mg/day for 24 weeks, and >500 patients were exposed to sitagliptin 100 mg/day for 52 weeks [34,38-44]. In phase III studies [34,40-42], adverse events were reported in 5% of patients treated with sitagliptin and were reported more than in patients who received placebo, regardless of causality. Such adverse events included upper respiratory tract infections (6.3%), nasopharyngitis (5.2%), and headaches (5.1%) [45]. The incidence of hypoglycemia with sitagliptin and placebo were comparable (1.2% of patients treated with sitagliptin and 0.9% given placebo) [46]. The prevalence of abdominal pain was 2.3% and 2.1% in sitagliptin and placebo arms, while the prevalence of nausea was 1.4% and 0.6% in sitagliptin and placebo arms, respectively [46]. Patients treated with sitagliptin demonstrated no significant increase in body weight from baseline [46].

A Cochrane review [47] of DPP-IV inhibitors found that all-cause infections (eg, nasopharyngitis, upper respiratory tract infection, urinary tract infection) showed a statistically significant increase after sitagliptin treatment (risk ratio 1.15, 95% CI 1.02-1.31; *P*=0.03). Furthermore, discontinuation due to adverse effects did not differ significantly between sitagliptin intervention and control arms. The risk ratios of serious adverse events did not show statistically significant differences between groups. The Cochrane review concluded that, overall, sitagliptin was well tolerated [47]. There is, however, no data on the adverse effects associated with long term use of sitagliptin.

Based on the mode of action of sitagliptin, it is plausible that the drug may reduce beta cell apoptosis and preserve beta cell functioning, thereby preventing the progression from prediabetes to type 2 DM. Animal and *in vitro* studies suggest that activation of GIP and GLP-1 receptors promotes beta cell resistance to apoptosis, proliferation, and neogenesis, resulting in enhanced beta cell function [37]. GLP-1 and GIP also promote beta cell proliferation and survival, and DPP-IV inhibitors exert similar effects in rodents with type 2 DM [48]. Sitagliptin prolonged islet graft retention in streptozotocin-induced diabetic mice [48]. Of the 56 studies that are currently investigating sitagliptin in diabetes, there are no studies investigating the effect of sitagliptin on the prevention of type 2 DM (Table 1).

**Table 1:** Summary of diabetes prevention studies.

Study	n	Study Arms	Duration	Endpoint	Results
Lifestyle modification and diabetes prevention					
The Finish Diabetes Prevention Study [18]	522	Lifestyle counselling, control group	3.2 years	Development of type 2 diabetes	Cumulative incidence of diabetes was 11% (95% CI 6-15%) in the intervention group and 23% (95% CI 17-29%) in control group
The Da Qing IGT and Diabetes Study [17]	577	Control group, diet only, exercise only, diet-plus-exercise	6 years	Development of type 2 diabetes	Cumulative incidence of diabetes at 6 years was 67.7% (95% CI 59.8-75.2) in control group compared with 43.8% (95% CI 35.5-52.3%) in diet group, 41.1% (95% CI 33.4-49.4) in exercise group and 46% (95% CI 37.3-54.7) in diet-plus- exercise group
Metformin and diabetes prevention					
The Diabetes Prevention Program Research Group [19]	3234	Placebo, metformin, lifestyle modification	2.8 years	Development of type 2 diabetes	Lifestyle intervention reduced incidence by 58% (95% CI 48-66%) and metformin by 31% (95% CI 17-43%), as compared to placebo
Ramachandran et al [22]	531	Control, lifestyle modification, metformin alone, lifestyle modification and metformin	30 months	Development of type 2 diabetes	Relative risk reduction 28.5% with lifestyle modification (95% CI 20.5-37.3%, *P*=0.018), 26.4% with metformin (95% CI 19.1-35.1, *P*=0.029), 28.2% with lifestyle modification and metformin (95% CI 20.3-37.0, *P*=0.022)
Thiazolidinediones and diabetes prevention					
The Diabetes Reduction Assessment with Ramipril and Rosiglitazone Medication [29]	5269	Rosiglitazone, placebo	3 years	Development of type 2 diabetes	Diabetes mellitus incidence in 49.5% of individuals in the rosiglitazone group (hazard ratio 0.40, 95% CI 0.35-0.46; *P*<0.001) and 69.7% in the placebo group (1.71, 1.57-1.87; *P*<0.001)
The Troglitazone in Prevention of Diabetes (TRIPOD) study [30]	133	Troglitazone, placebo	30 months	Development of type 2 diabetes	Average annual diabetes incidence rates in women who returned for follow up were 12.1% and 5.4% in placebo and troglitazone groups, respectively The hazard ratio for diabetes was 0.45% (95% CI 0.25-0.83) in the control group and 0.50 (95% CI 0.28-0.89) in the troglitazone group
The Pioglitazone in Prevention of Diabetes (PIPOD) study [31]	95	Pioglitazone, placebo	3 years	Development of type 2 diabetes	Average annual incidence rates of diabetes were 5.2% during pioglitazone treatment and 4.6% during the entire observation period, including the post-drug washout The final cumulative incidence of diabetes during treatment and post drug follow up was 17%

### Motivation for the Study

The high global prevalence of type 2 DM, and its associated morbidity and mortality, place major demands on health care resources (both human and financial). Developing countries are faced with a high prevalence of both infectious diseases and diseases of lifestyle. Reducing the incidence of type 2 DM will reduce the demand on limited health care resources.

Type 2 DM is predated by a condition known as prediabetes, which offers an opportunity for targeting preventative measures. There is currently great interest in the search for interventions to prevent type 2 DM. Various pharmacological and nonpharmacological agents have been used with various degrees of success. Since DPP-IV inhibitors and biguanides have differing pharmacological modes of actions, we propose that combining these agents may have additive and possibly synergistic effects on preventing the progression from prediabetes to type 2 DM. The combination of the aforementioned drugs will allow for the reduction in the prescribed doses of each agent, and thus may limit the probability for adverse drug effects.

Sitagliptin is a novel antidiabetic agent that theoretically possesses the ability to preserve existing beta cell function by preventing beta cell apoptosis, and also increases beta cell mass. These effects have been shown *in vitro* and in animal studies. Furthermore, current clinical data indicate that sitagliptin is safe in the short term. This will be the only study investigating the effect of the combination of sitagliptin and metformin on prediabetes progression.

### Developing Country Dynamics and Clinical Trials

Conducting a trial of this magnitude in a developing country encompasses numerous challenges, including the availability of human and financial resources. Clinicians involved in this study will be those that are currently in training or employed in the public sector, and are affiliated with a teaching hospital and medical university. The trial will also allow for the exposure of medical doctors to clinical trials, and will allow for their training in good clinical trial practice.

The use of resources for the prevention of type 2 DM is an opportunity cost for HIV/AIDS and other chronic disorders. However, we propose that resources spent in the short term for diabetes prevention may, in the long term, allow for more resources to be allocated to competing disease conditions.

Currently, we plan to obtain funding from the pharmaceutical industry, endocrine societies, University of Witwatersrand, University of Cape Town, University of Pretoria, Medical University of South Africa, University of KwaZulu-Natal, and South African Department of Health. The trial will be registered on the National Institutes of Health (NIH) clinical trial database, subsequent to approval by the university ethics review boards and confirmation to the regulations of National Health Authority.

### Limitation of the Study

The study is only 5 years in length, and thus cannot truly determine the effect of interventions on progression from prediabetes to type 2 DM.

## Methods

The aim of the study is to determine the effect of sitagliptin and metformin on progression from prediabetes to type 2 DM.

### Objectives

This study has seven primary objectives, namely to determine: (1) the effect of metformin and placebo on glycemic endpoints; (2) the effects of sitagliptin and metformin on glycemic endpoints; (3) the effects of metformin and placebo on incidence of cardiovascular disease and death; (4) the effects of sitagliptin and metformin on incidence of cardiovascular disease and death; (5) the incidence of adverse effects associated with metformin and placebo; (6) the incidence of adverse effects associated with sitagliptin and metformin; and (7) the quality of life (QOL) of subjects using metformin and sitagliptin.

### Study Population

The study population will consist of subjects referred from *peripheral sites* within Johannesburg, Pretoria, Durban, and Cape Town. These *peripheral sites* will include general practitioners, primary health care clinics, and other facilities in which screening glucose tests are performed. High risk subjects will be screened for IGT. Subjects with high risk for prediabetes, in whom screening may be warranted, include the following groups: age >45 years and overweight (BMI >25 kg/m^2^); cardiovascular events (eg, myocardialinfarction); age <45 years and overweight with a first degree relative with DM, previous gestational diabetes or macrosomia in one or more children, or have hypertension or dyslipidaemia; patients of Asian descent with a lower BMI (>23 kg/m^2^); and patients with thyroid dysfunction.

### Study Timeline

#### Visit 1 - Study Start

Subjects referred from *peripheral sites* to the study sites will be briefed about the study and invited to participate. Thereafter, informed consent will be obtained. Appropriate tests will be performed to determine if subjects meet the criteria for inclusion in the study (eg, liver function tests [LFTs], urea and electrolytes, HbA1c, FBG, fasting blood insulin [FBI] level, physical examination, urate levels, and blood gases for pH determination). See Textbox 1 for inclusion, exclusion, and withdrawal criteria for the study. Drug history will be obtained and anthropometric measures determined. Subjects will be told that they must return the following week for their results and for appropriate counseling.

**Inclusion, exclusion, and withdrawal criteria for the study.**

**Inclusion Criteria**

- Informed consent
- Subjects with impaired glucose tolerance as defined by American Diabetes Association
  - Impaired glucose tolerance (2-hour postprandial glucose of 7.8–11.0 mmol/L)
  - Impaired fasting blood glucose (fasting glucose of 5.6-6.9 mmol/L)
- Age 18-65 years
- No history of liver disease
- Negative pregnancy test

**Exclusion Criteria**

- Impaired liver function tests
- Cardiac failure or history of congestive heart failure in the close family
- Medication that may affect insulin resistance (eg, oral hypoglycemic agents, thiazide diuretics)
- Contra-indications to exercise
- Pregnancy
- Patients planning to move residence within the next 5 to 10 years
- History of hypersensitivity reaction to sitagliptin, such as anaphylaxis or angioedema

**Withdrawal Criteria**

- Withdrawal of informed consent
- Congestive cardiac failure
- Impaired liver function
- Lactic acidosis
- Clinical or biochemical evidence of hypoglycemia
- Drug usage should be temporarily discontinued in patients undergoing radiologic studies involving intravascular administration of iodinated contrast materials, because use of such products may result in acute alteration of renal function
- Excessive rapid weight gain, dyspnea, and/or edema
- Renal disease or renal dysfunction
  - Serum creatinine levels >1.5 mg/dL (males), >1.4mg/dL (females)
  - Abnormal creatinine clearance, which may also result from conditions such as cardiovascular collapse (shock), acute myocardial infarction, and septicemia
- Acute or chronic metabolic acidosis, including diabetic ketoacidosis, with or without coma
- Pregnancy

#### Visit 2 - 2 Weeks Later

Subjects meeting the inclusion criteria will be invited to join the study. Study subjects not meeting the inclusion criteria will be counseled and a detail letter will be sent to the initial referring site for further treatment. Patients unable or unwilling to return to their initial referring site will be treated at the study sites. All subjects will be given dietary advice and a standard exercise protocol from the Sports Science Department, and will be allowed access to the university gym. Subjects will be randomized to either metformin extended release (500 mg daily) and placebo (daily), or metformin extended release (500 mg daily) and sitagliptin (25 mg daily), for one month.

#### Visit 3 - 2 Weeks Later

Measures for determining safety and measures of glycemic control will be examined. The dose of metformin and sitagliptin will be increased to 1000 mg and 50 mg daily, respectively (provided that the patients have tolerated the initial trial of drug). Subjects who have progressed to type 2 DM (based on indicators of glycemia such as FBG) will be referred to the diabetic clinic for management. LSM advice will be reenforced.

#### Visit 4 - 1 Month Later

Measures of glycemia (FBG and HbA1c) will be repeated, along with biochemical and clinical tests for safety. Anthropometric measures, lipid profiles, QOL forms, and FBI levels will also be examined.

#### Visits 5 Through 25

Every two months, the parameters examined in Visit 4 will be repeated, until the 1-year time point after study initiation. Subsequent study visits will occur every three months and the investigations will be repeated. A total of 25 visits will occur over a period of 5 years. During the second last visit, all trial medication will be stopped and subjects will return to the clinic two weeks later for further assessment. Measures of glycemia, LFTs, renal function, anthropometric measures, lipid profiles, QOL forms, safety data (biochemical and clinical), and FBI levels will all be assessed. An outline of the study assessments and visits are contained in Table 2 and Table 3, respectively.

**Table 2:** Study assessment during various visits.

	Visit number																									
**Assessments**	1^a^	2	3	4	5	6	7	8	9	10	11	12	13	14	15	16	17	18	19	20	21	22	23	24	25
**Hematology**	X	X	X	X	X	X	X	X	X	X	X	X	X	X	X	X	X	X	X	X	X	X	X	X	X
**Liver function test**	X	X	X	X	X	X	X	X	X	X	X	X	X	X	X	X	X	X	X	X	X	X	X	X	X
**Urea and electrolytes**	X	X	X	X	X	X	X	X	X	X	X	X	X	X	X	X	X	X	X	X	X	X	X	X	X
**Glycosylated hemoglobin**	X	X	X	X	X	X	X	X	X	X	X	X	X	X	X	X	X	X	X	X	X	X	X	X	X
**Fasting blood ** **glucose**	X	X	X	X	X	X	X	X	X	X	X	X	X	X	X	X	X	X	X	X	X	X	X	X	X
**Fasting blood insulin**	X	X	X	X	X	X	X	X	X	X	X	X	X	X	X	X	X	X	X	X	X	X	X	X	X
**Clinical examination**	X	X	X	X	X	X	X	X	X	X	X	X	X	X	X	X	X	X	X	X	X	X	X	X	X
**Anthropometric measures**	X	X	X	X	X	X	X	X	X	X	X	X	X	X	X	X	X	X	X	X	X	X	X	X	X
**Adverse events**	X	X	X	X	X	X	X	X	X	X	X	X	X	X	X	X	X	X	X	X	X	X	X	X	X

^a^Screening after documented informed consent obtained

**Table 3:** Study time period and study procedures.

Visit	Time interval	Study procedures	Cumulative time period
1	Study start	Patients referred from peripheral sites Patients interviewed and informed consent obtained Tests to determine if subjects fulfil inclusion criteria Drug history and anthropometric measures Patients informed to attend next visit after one week for results and appropriate counselling	0
2	2 weeks	Subjects fulfilling inclusion criteria will be invited to partake in the study Subjects not fulfilling the inclusion criteria will be counselled and directed back to initial referral center All subjects will be given dietary advice and a standard exercise protocol, and will be allowed access to the university gym Randomized to either metformin (500 mg daily) and placebo (daily) or metformin 500 mg daily and sitagliptin (25 mg daily) for one month	2 weeks
3	2 weeks	Efficacy and safety measures Dose escalation to metformin 1000 mg and sitagliptin 50 mg daily Subjects that have progressed to type 2 DM will be referred for management Lifestyle modification advice reenforced	1 month
4	1 month	Glycemic measures repeated Tests for safety (biochemical and clinical), anthropometric measures, lipid profiles, quality of life forms, safety data, FBI levels	2 months
5	2 months	Repeat	4 months
6	2 months	Repeat	6 months
7	2 months	Repeat	8 months
8	2 months	Repeat	10 months
9	2 months	Repeat	1 year
10	3 months	Repeat	1 years and 3 months
11	3 months	Repeat	1 years and 6 months
12	3 months	Repeat	1 years and 9 months
13	3 months	Repeat	2 years
14	3 months	Repeat	2 years and 3 months
15	3 months	Repeat	2 years and 6 months
16	3 months	Repeat	2 years and 9 months
17	3 months	Repeat	3 years
18	3 months	Repeat	3 years and 3 months
19	3 months	Repeat	3 years and 6 months
20	3 months	Repeat	3 years and 9 months
21	3 months	Repeat	4 years
22	3 months	Repeat	4 years and 3 months
23	3 months	Repeat	4 years and 6 months
24	3 months	Repeat and medication stopped	4 years and 9 months
25	3 months	Glycemic measures and safety tests Patients referred to appropriate clinics	5 years

### Blinding

Blinding will be achieved by formulating a product that is identical to sitagliptin in appearance, but does not contain the pharmacologically active agent *.*

### Endpoints

Primary endpoints include the number of subjects progressing from prediabetes to type 2 DM, the number of cardiovascular events, and the number of deaths. Secondary endpoints include lipograms, urea and electrolytes, LFTs, full blood count, FBG, FBI, weight and other anthropometric parameters, and blood pressure.

### Safety Considerations

Pharmacological agents are not without adverse effects, and in designing this study the probability and severity of adverse effects were considered. The safety of the interventions was very important due to the long duration of the study. We thus had to ensure the inclusion of agents that were pharmacologically rational and safe. Metformin has been used for many decades and is relatively safe, and we have developed inclusion criteria to ensure that subjects susceptible to lactic acidosis (a rare but serious adverse effect of metformin) would be excluded from the study. Sitagliptin has been used for the therapy of type 2 DM and has been associated with minimal adverse effects. This drug has only been on the US market for approximately six years and has proven safe thus far. The current study will have a duration of 5 years, and the frequent evaluation and close monitoring over the 5-year study period will enable monitoring of any serious adverse effect.

Safety measures have been incorporated into the study, including LFTs, renal function tests, and the regular clinical evaluation of patients for adverse effects. Subjects suspected of having study-drug related adverse effects will be aggressively investigated and managed at the cost of study team. Furthermore, we will monitor the use of sitagliptin for diabetes in the global market, with particular note of its adverse effect profile. If the safety benefit ratio of sitagliptin becomes unacceptable, the study will be stopped. To further augment safety, we will use low doses of both sitagliptin and metformin.

### Ethical Considerations

The study protocol will be submitted to the ethics committee at the University of the Witwatersrand. Permission to conduct the study at public sector health care sites will be obtained from the managers of the named institutions, and the Director General of Health in the provinces in which the study is to be conducted. The study will be conducted in accordance with the Declaration of Helsinki [49] and its amendments, and the Patients’ Rights Charter. Subjects will be asked to provide written informed consent to participate as a criterion for entry into the study.

### Value of the Study

This study aims to determine the efficacy of pharmacotherapy in preventing the progression from prediabetes to type 2 DM, and thus may add to the armamentarium of agents utilized for the management of prediabetes.

### Data Management

The clinicians at the study sites will fill out all study forms. Study coordinators at the various study sites will check the forms for completeness. The data manager will then also check all forms for completeness and enter the data onto a database. Lists of the subjects who need to be called back will be printed by the data manager, and faxed and emailed to the study sites to ensure that study participants are reminded of their study visit dates and times. Confidentiality will be maintained by allocating a code number to each participant, and original data collection forms for each patient will be kept safe and strictly confidential.

### Statistical Analysis

This study will compare two groups, one of which will receive metformin and sitagliptin, and the other metformin and placebo. The primary end point is progression from prediabetes to type 2 DM at the end of the 5-year study period. Sample size, determined by a statistician, is based on the following assumptions: the rate of development of type 2 DM will be, at most, 50% after a 5-year follow-up in the group that receives placebo and metformin; the rate of development of type 2 diabetes will be 30% (20% reduction) after a 5-year follow up in the group that receives sitagliptin and metformin; and at most, the dropout rate of participants will be between 10-20% per year.

For a 5% significance level and 90% power, 134 participants are required in each group (268 participants total) at the end of the study. This value translates to between 228 and 410 participants in each group at the beginning of the study to allow for 10-20% loss to follow-up in each year. We thus chose to include 410 subjects in each group. Data analyses will include clinically relevant measures (eg, numbers needed to treat and numbers needed to harm) and will be done according to the intention-to-treat principle.

## Results

This study is currently in the funding phase.

## Discussion

The high morbidity and mortality associated with type 2 DM [2,4-6,50], and its ability to consume health care resources, make it an important target for primary prevention [51]. Various studies [17-19] have demonstrated that lifestyle intervention is effective in preventing type 2 DM. However, lifestyle interventions comparable to those used in the aforementioned studies would require significant investments by the subject and the community [14,52,53]. Adherence to lifestyle interventions in clinical trials, in which subjects are given extensive support, is generally poor [52,53]. Medication, although less effective than LSM, may have the added benefit of improved compliance. Valensi et al [8] in their European Consensus statement, recommend that pharmacological intervention combined with diet and exercise counselling may be the most realistic option for achieving real reductions in diabetes incidence.

Ameliorating insulin resistance could influence the progression from IGT to type 2 DM. The combination of a biguanide with a thiazolidinedione is pharmacologically rational [32-34] since these agents target insulin resistance via different mechanisms. However, this combination does not address beta cell dysfunction, which is an important factor in the progression from prediabetes to type 2 DM. Currently, the literature does not contain the results of any trials investigating the effect of combining a biguanide and thiazolidinedione on the progression of prediabetes to type 2 DM. However, the NIH has registered a clinical trial that is investigating the effect of combining rosiglitazone and metformin to determine their effects on individuals with IGT. Rosiglitazone has more adverse effects compared to pioglitazone, leading to our hypothesis that the combination of pioglitazone and metformin may be associated with fewer adverse effects. The current study registered in the NIH clinical trial database is not blinded or randomized, thus reducing its quality. However, poor publicity, and the association of the thiazolidinediones with fatal hepatic failure [54], was a deterrent to their use. Furthermore, having three study arms meant that a greater number of patients would need to be recruited into the study, thereby increasing the resources required. Finally, motivating patients to use a combination of drugs for a disease that they do not actually have, with a drug that has been associated with life-threatening adverse effects [54], was unacceptable and ethically unjustifiable. As such, we decided to exclude the combination of thiazolidinedione and biguanide, based on an unacceptable safety benefit ratio when considered for prediabetes.

The development of DPP-IV inhibitors has added to the armamentarium of pharmacological agents available for the treatment of type 2 DM [36]. The efficacy of these drugs in type 2 DM treatment compares favorably to other oral antidiabetic agents [47]. Sitagliptin, an orally administered DPP-IV inhibitor, has been shown to preserve beta cell function [37], thus having the theoretical potential to prevent the progression from prediabetes to type 2 DM. This clinical trial will determine whether the promising results in animal studies will translate to clinical utility.

The combination of sitagliptin and metformin is pharmacologically rational since each drug has a different mode of action and good safety profile [23-27,34,36,40-42,45-47]. This combination will target both insulin resistance and beta cell dysfunction, which are key pathological hallmarks of prediabetes. We postulate that the beneficial effects of this combination on prediabetes will be greater than that of metformin alone. Further rationale for combining metformin with sitagliptin supposes that patients using this combination will have the benefit of proven metformin efficacy [28], and further possible protective effects of sitagliptin. Furthermore, this approach enables us to determine the potential benefit of this untested combination.

Blinding is not always practical, and clinicians can sometimes determine which therapy is which (ie, *break the code)*. Blinding in this study will be accomplished by using metformin in both arms, and having sitagliptin in one arm and a preparation with the appearance of sitagliptin (but without the active ingredient) in the other arm. This tactic will make *breaking the code* more difficult, thereby limiting potential for bias.

This study is designed to include clinically relevant endpoints, and the strength of the study design will facilitate appropriate conclusions. The study is designed to include blinding, intention-to-treat analysis, and randomization in a homogenous population. The study has a sufficient follow-up period and uses clinically relevant parameters to determine the magnitude of the treatment effect (control event rate, experimental event rate, relative risk reduction, absolute risk reduction, and numbers needed to treat). The precision of the estimate of treatment effect will be gauged by the calculation of confidence intervals. Furthermore, the applicability of the results to patients with prediabetes encountered in clinical practice was also considered; in doing so, we considered whether patients in clinical practice would be similar to those in our study, feasibility of treatment in our setting, and potential benefits and harms. Consequently, this study is in keeping with the trend to design clinical trials to ensure conformity to evidence-based medicine.

Conducting this study in a resource-poor setting is challenging. The cost of bringing many experts together and harnessing their skills is high, so we chose to make the study a collaboration in which experts throughout the country would be engaged. The incentives for these experts to join the study are that (1) they will apply their knowledge to answering a clinically relevant study question for not only South Africa and Africa, but the entire world, and (2) they will share authorships in the publications, provided that they make contributions that will make them eligible for coauthorship. Most importantly, we envisage that the multidisciplinary team will improve the quality of the study. Diabetes is a disease that requires a multidisciplinary approach and this study will require the expertise of individuals from diverse fields, including internal medicine, endocrinology, sports science, pharmacology, diabetes, chemical pathology, psychology, and biostatistics. A coordinated team approach will harness individual strengths to help build a team of experts that will propel the study in the face of financial and human resource challenges.

Another challenge of the study is that the interventions in this study may also control glycemia and thus *mask* the biochemical evidence of type 2 DM. One method of overcoming this limitation would entail the use of washout periods at regular intervals. However, this approach would be resource-intensive and inconvenient to study participants, as it would interrupt the routine and may jeopardize compliance. Based on this consideration, we decided to have a single washout period at the end of the study period to determine how many subjects have actually become diabetic.

Evidence favoring the use of LSM and holistic approaches to the treatment of diabetes and prediabetes has inspired us to include LSM in both arms of the study. Furthermore, the proven efficacy of metformin in prediabetes has influenced our decision to include this agent in both of the study arms, thus allowing study participants the full benefit of the best current evidence-based practice. The addition of sitagliptin to the metformin arm attempts to take advantage of the beta cell sparing effects of sitagliptin, and it is hoped that this combination will have greater effects than metformin alone. The exclusion of the thiazolidinedione drugs is based on their poor safety record and the long duration of the study (even relatively mild adverse effects over a protracted period of time may compromise compliance). It was paramount to ensure that study subjects received safe drugs to ensure a favorable safety/risk benefit.

In summary, DM is associated with high morbidity and mortality that places major demands on health care resources. It is important to reduce the incidence of type 2 DM by preventing progression from prediabetes to diabetes. LSM remains the gold standard to prevent progression from prediabetes to diabetes, but adherence to LSM is challenging, even in the controlled environments of clinical trials. This study investigates the potential of a low dose combination of a biguanide (metformin) and DPP-IV inhibitor (sitagliptin) to prevent progression from prediabetes to type 2 DM. The choice of the aforementioned pharmacological combination is based on good safety profiles for each drug, and their complementary modes of action.

## Abbreviations

BMI: body mass index
CANOE: Canadian Normoglycemia Outcomes Evaluation
DM: diabetes mellitus
DPP-IV: dipeptidyl peptidase-IV
FBG: fasting blood glucose
FBI: fasting blood insulin
GIP: glucose dependent insulinotropic peptide
GLP-1: glucagon-like peptide 1
HbA1c: glycosylated hemoglobin
IGT: impaired glucose tolerance
LFT: liver function tests
LSM: lifestyle modification
NIH: National Institutes of Health
PIPOD: Pioglitazone in Prevention of Diabetes
QOL: quality of life
SD: standard deviation
SiMePreD: Sitagliptin and Metformin in PreDiabetes
TRIPOD: Troglitazone in Prevention of Diabetes
